# A Comparison of Mere-Exposure, Flavor–Flavor and Flavor–Nutrient Learning Strategies in Enhancing Legume Preferences in an Adult Sample

**DOI:** 10.3390/foods15162884

**Published:** 2026-08-18

**Authors:** Isabella Tao Jakobsen, Derek V. Byrne, Barbara Vad Andersen

**Affiliations:** 1 Food Quality Perception and Society Team, iSense Lab, Department of Food Science, Faculty of Technical Sciences, Aarhus University, 8000 Aarhus, Denmark; derekv.byrne@food.au.dk (D.V.B.); barbarav.andersen@food.au.dk (B.V.A.); 2 Sino-Danish Center for Education and Research, University of Chinese Academy of Sciences, Beijing 101408, China

**Keywords:** eating behavior, sustainability, consumer, sensory learning, legumes

## Abstract

Shifting dietary patterns is necessary to meet global environmental and health targets. Legumes represent a nutritious and viable protein source. Yet, consumption remains low among consumer groups due to low familiarity and inferior sensory perceptions. Sensory conditioning has proven effective in increasing acceptance of unfamiliar foods and may have applicability in shaping future legume preferences. The study compared Mere-Exposure, Flavor–Flavor and Flavor–Nutrient learning strategies in their effectiveness in increasing consumer preferences for legumes in a Danish adult sample (*N* = 70). Participants filled out a questionnaire before and after a three-week at-home meal intervention. Fava beans were used as the legume case, and all participants, were exposed to 12 fava bean test stimuli. Participants in the Mere-Exposure group experienced a significant increase in preferences, while the Flavor–Flavor and Flavor–Nutrient groups did not. The Mere-Exposure group also experienced an increase in liking of the appearance and flavor of fava beans. By showcasing the potential of a widely applicable sensory strategy, the study highlights how sensory conditioned learning might support the adoption of more sustainable foods in an adult sample. The results provide insights for health and nutritional personnel, researchers and policymakers seeking to promote sustainable diets.

## 1. Introduction

Numerous studies support the health and environmental benefits of consuming a diet rich in plant-based food sources. Legumes, in particular, provide essential proteins, making up to 30% of their dry weight, along with dietary fiber, and important micro- and phytonutrients. Their nutritional composition supports optimal human health and has been associated with improved dietary quality, cardiovascular and gastrointestinal health benefits, while supporting a shift towards more sustainable farming practices [1,2,3,4,5,6].

Legumes are among the most important food crops globally and contribute substantially to food security due to their widespread cultivation and high nutritional value [1,4,6]. Due to this, legumes are included in a wide array of official nutritional guidelines in different countries [7,8,9,10,11,12].

However, while global legume consumption has seen some increase, progress is insufficient to meet dietary and sustainability targets and is marked by substantial between-country variability [9,13]. Legume consumption is particularly low in European or westernized countries, and collectively, these patterns highlight a gap between policy ambitions and dietary practices [10,11,12,13,14]. A recent national dietary survey from the DTU National Food Institute in Denmark found that only 0.8% of the Danish population complies with the national dietary recommendations of 100 g of legumes daily [14], showcasing that there is substantial room for improvement. To put it into the words of Foyer et al. [15]: “Neglecting legumes has compromised human health and sustainable food production”.

The compromised benefits are believed to be related to several factors which hinder an increase in legume consumption, such as preparation difficulties, a lack of familiarity, and lower acceptability amongst consumers on account of inferior perceived palatability [16,17,18,19,20,21,22]. Additionally, a recent cross-cultural study found that the surveyed consumers had low motivation to consume legumes and that the role of preference formation regarding legumes was poorly understood [23]. In general, a desire for tastiness prevails as the main driver of food choice [24,25], and seems significant for legume preferences as well [19,21,26]. Consumer preferences are though continuously evolving and should not be underestimated.

### 1.1. Development of Food Preferences

Turning towards the development of food preferences, humans have both a genetic predisposition towards selecting certain tastes as well as acquired preferences. Humans display a strong predisposition for sweet taste at birth [27], and a preference for salty taste is observed from the age of four to five months [28]. Bitter and sour tastes are often rejected [29]; however, an age gradient is observed depending on the component tested. These specific taste preferences and aversions are believed to be evolutionary adaptations. Sweetness signals energy-rich carbohydrates, which were vital for survival in ancestral environments, and to secure intake of the first human food, breast milk. Salt is essential for maintaining electrolyte balance, nerve function, and hydration [30,31]. On the contrary, many toxic plants and substances taste bitter, and sour can signal spoilage, so these aversions are thought to serve protective evolutionary mechanisms [32]. Regardless of the many benefits of legume consumption, legumes can produce off-flavors such as bitterness and astringency, which contribute to negative sensory perceptions and lower acceptability [33]. Thereby, legume distaste may be related to our evolutionary flavor adaptations.

Despite our inherent predisposition for certain tastes, human preferences are not invariably fixed but can be adapted through repeated or associative food experiences. Consistent exposure to specific foods shapes our preferences, showcasing how familiarity can increase dietary variety [34]. Pavlovian associative learning has been suggested as a plausible explanation for the acquisition of food likes and dislikes in humans [35]. It posits that the evaluative value of foods may be influenced by stimuli presented consistently along with the foods in question. Similarly, sensory learning stems from Pavlovian conditioning and is based on the premise of affective conditioning, while building on the influences of conditioned and unconditioned stimuli [35]. In the context of food preference formation, conditioned stimuli refer to target stimuli that an individual needs to learn to like, while unconditioned stimuli refer to a type of stimulus that is already liked.

### 1.2. Sensory Strategies to Affect Food Preferences

Three primary learning processes fall under sensory conditioning, namely, Mere-Exposure, Flavor–Flavor and Flavor–Nutrient learning [36,37,38]. The sensory conditioned learning strategies differ in their mechanisms but are all relevant when attempting to affect food preference; i.e., increase preference for a certain food. Mere-Exposure learning assumes that repeated exposure to a target stimulus will positively affect an individual’s attitude towards it [38]. Researchers have extensively investigated and confirmed the Mere-Exposure effect, i.e., that humans increase liking towards a stimulus as it becomes more familiar [39,40,41,42,43]. Flavor–Flavor learning [36,44,45] refers to the process where the preference of a conditioned stimulus, when paired with an unconditioned stimulus, develops in the direction of the unconditioned stimulus. When the unconditioned stimulus is liked, it may result in a positive attitudinal response towards the conditioned stimulus via associative learning. This implies that when combining a disliked or neutral food with a well-liked flavor, the food becomes increasingly liked through association, even after the well-liked flavor is removed. Flavor–Nutrient learning is based on the premise that an individual can develop a preference for an initially disliked, neutral or unfamiliar food if it is consistently paired with a caloric or nutrient-dense reward. In terms of the underlying associative learning theory, in Flavor–Nutrient learning, the conditioned stimuli becomes associated with the positive post-ingestive sensation of the unconditioned stimuli [37,46], and liking for the disliked, neutralor unfamiliar food increases gradually in the direction of the positive post-ingestive reward, even after the reward is removed.

The three strategies are well established, with Mere-Exposure learning consistently emphasized as the most efficient in increasing acceptance of novel flavors and foods [42,47,48]. While Flavor–Flavor learning demonstrates several successes, an equal number of failures to detect these effects have been found [48]. The effects of Flavor–Nutrient learning are effectively documented in animal studies, while the effects are more elusive in humans [49].

### 1.3. Affecting Adults’ Preference for Legumes via Sensory Learning Strategies

Previous sensory conditioning studies have mainly focused on children or adolescents, vegetable consumption, disordered eating in adults or health promotion [42,47,50,51,52,53,54,55], whereas little focus has been placed on utilizing sensory conditioning to shift adult consumers towards more sustainable eating behaviors. A comparison of Mere-Exposure, Flavor–Flavor and Flavor–Nutrient learning and their efficacy in shifting sensory modalities in adults is likewise limited. Clarifying the effect of these strategies among adults is especially relevant for sustainable eating behavior transitions, where resistance to change is often linked to poor sensory expectations and eating habits [16,17,21,56,57,58,59]. Compared to children, adults often demonstrate lower learning flexibility and more established food preferences, making behavior change a complex challenge [56,58,60]. While supporting sustainable eating behavior in children is equally important, adult consumers differ in terms of their cognitive and behavioral drivers of food choice [61,62]. Furthermore, whereas children’s food choices are heavily influenced by parental control and the home environment [63], adults are responsible for most independent food consumption and purchasing decisions and directly impact food-system demand. Adults, therefore, represent a particularly important population for investigating the comparative effect of sensory learning strategies. Much of the existing evidence has focused on children and adolescents [39,40,42,47,50], highlighting the need for studies investigating the effectiveness of Mere-Exposure, Flavor–Flavor and Flavor–Nutrient learning strategies in adult study populations. Establishing whether these sensory learning mechanisms can remain effective in adulthood is important for understanding their applicability in supporting more sustainable dietary transitions. The comparison of how Mere-Exposure, Flavor–Flavor and Flavor–Nutrient learning affect consumer preferences of legumes is of both theoretical and practical relevance, adding to the body of literature on how to shift adult consumers towards more sustainable eating behaviors. Grounded in the theory of Mere-Exposure, Flavor–Flavor and Flavor–Nutrient learning, the present study was developed with a specific focus on comparing preference conditioning pathways for legumes. The reasoning for this case study is that legumes present a critical possibility within sustainable nutrition. Legumes offer a widely available nutrient source, but are hindered by their currently low intake, and consumers’ taste and texture aversions towards them and legume based foods [64,65]. Thus, legumes serve as an appropriate food source to investigate how sensory conditioned learning strategies may or may not influence adult sustainable food preferences.

#### Aim and Hypothesis

The current study aimed to address two aims: (1) to compare the effect of Mere-Exposure, Flavor–Flavor and Flavor–Nutrient learning on adult consumers’ preference for an unfamiliar legume (fava beans), and (2) to examine whether a potential effect could be transferred to a different legume (butter beans). Based on the literature, it was hypothesized that the Mere-Exposure strategy would increase preference for legumes beyond the Flavor–Flavor and the Flavor–Nutrient earning strategies.

## 2. Materials and Methods

### 2.1. Overall Study Design

**Figure 1:**
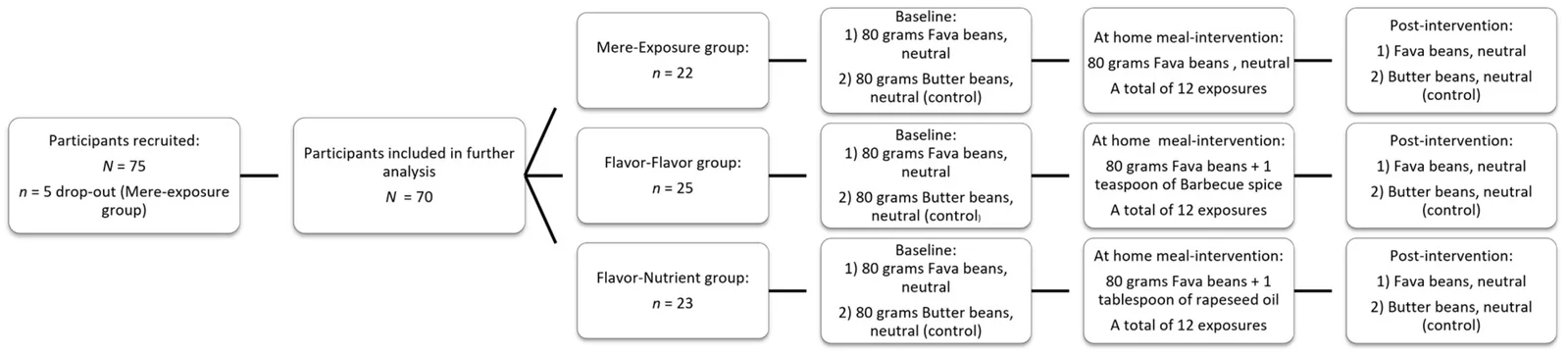
Illustration of the experimental design, points of measurements and test-meals. Participants’ preference for neutral fava bean (test stimulus) and butter bean (control stimulus) were tested at baseline and post-intervention. The post-intervention measurement followed an exposure period, where participants were divided into three intervention groups: participants in the Mere-Exposure group were exposed to neutral fava beans, the Flavor–Flavor group to barbeque-spiced fava beans, and the Flavor–Nutrient group to rapeseed oil-added fava beans. The intervention period was 12 exposures over 3 weeks at home, in all groups.

The study utilized an independent measures design, where a total group of naïve consumer participants (*N* = 70) was divided into three intervention groups, reflecting the sensory conditioned learning strategies of Mere-Exposure (*n* = 22), Flavor-Flavor (*n* = 25) and Flavor-Nutrient learning (*n* = 23). The study applied a within-subject repeated measures structure within each group, with two primary timepoints for measurements: a baseline measurement and a post-intervention measurement, with the latter conducted after 12 exposures to the target legume (Figure 1). This allowed for a direct comparison of groups, as each consumer completed every data point. Data were collected at baseline and post-intervention via questionnaires. Naïve consumers were chosen as the most appropriate target population to include for affective preference measurements. The study design of the current study aligns with previous studies comparing Mere-Exposure, Flavor–Flavor and Flavor–Nutrient learning strategies [42].

### 2.2. Recruitment, Participants and Exposure Groups

A total of 75 participants were recruited through the recruitment sites Sona (Aarhus University) and TrialTree, convenience sampling, snowballing, and social media (Facebook^®^, LinkedIn^®^). Recruitment procedures were aimed at including a broad demographic that could represent a subset of the larger consumer base of legumes. Participants meeting the following criteria: ≥18 years old, healthy (self-assessment), moderate preference for legumes (self-assessment, from 1: Do not like at all to 10: Like extremely much), Danish language proficiency, and a lack of familiarity with the target legume (fava beans) were considered eligible for the study. All potential participants were screened via an initial phone call prior to participation in the study. The participants were considered adults if they were ≥18 years old; no age limitation was applied for the participants as long as they were mentally and physically fit to participate.

The 75 participants were divided into three groups: A Mere-Exposure group, a Flavor–Flavor group and a Flavor–Nutrient group, emphasizing an equal gender balance between groups and balanced pre-study preference towards legumes in general (Table 1). The participants were recruited continuously and assigned to one of the three intervention groups using a gender-stratified allocation procedure to achieve a comparable distribution of men and women across groups. Because the Flavor–Flavor intervention relied on a positive perception of the conditioned stimulus, only participants reporting a liking score of ≥8/10 for barbecue spice during initial screening were eligible for this group. No additional allocation criteria were applied to the Mere-Exposure or Flavor–Nutrient groups. The baseline participant characteristics in Table 1 were collected after intervention group allocation and were therefore not used in the assignment of participants to intervention conditions for the Mere-Exposure, Flavor–Flavor and Flavor–Nutrient groups.

During the study, 27 participants were initially recruited to the Mere-Exposure group. However, five participants dropped out, resulting in the final inclusion of *n* = 22 participants in the Mere-Exposure group. No participants dropped out of the Flavor–Flavor group (*n* = 25) nor the Flavor–Nutrient group (*n* = 23), and 70 participants were included in total in the further analyses. Given the exploratory nature of the study, the sample size was deemed appropriate, as it falls within the range reported in a systematic review of food exposure and conditioning interventions of approximately 20 participants per group [39]. Power was calculated for *N* = 70, α = 0.05 and a statistical power of 0.80. The minimum detectable effect size was Cohen’s f = 0.38, corresponding to a medium-to-large effect. Consequently, the study was considered sufficiently powered to detect moderate-to-large group differences, whereas smaller effects may have remained undetected.

### 2.3. Experimental Procedure and Measurements

Prior to initiation, the study was ethically approved by Aarhus University’s Research Ethics Committee, journal number: 2025-0809265, and written consent was obtained from all the participants. All participants were paid for their participation, according to the regulations for research participation at the Cognition and Behavior Lab (COBE Lab), Aarhus University. The study consisted of a baseline and post-intervention measurement conducted at a central lab location, and an in-between intervention period, conducted in the natural setting of the participants’ daily life.

Baseline measurements: the measurements were conducted on-site at Agro Food Park or COBE Lab, Aarhus University. Participants were instructed not to eat two hours before the baseline measurement to facilitate matching appetite levels. Upon arrival, participants were given oral and written information about the study, focusing on the procedure of accessing the questionnaire (via a QR code, on the participants’ phones), and following the instructions in the questionnaire about consumption of the test-meals and answering questions. First, all participants answered questions in Sections 1 and 2 of the questionnaire (refer to Section 2.5). Then, they received the primary test stimulus (fava beans, neutral) and answered the related preference questions in questionnaire Sections 3–5. To conclude, the participants received the control test stimulus (butter beans, neutral) and answered the related preference questions in the questionnaire (see Questionnaire Section 2.5).

Intervention period: the intervention period was conducted in the natural setting of participants’ daily life to maximize the ecological validity of the study. Each participant was given written instructions specifying the standard preparation procedure, amount of fava beans, additional ingredients to be added (for the Flavor–Flavor and Flavor–Nutrient groups only), and the exact number of exposures to consume during the intervention. The participants were instructed to consume a total of 12 legume test-meals during the 3-week intervention period. To minimize potential effects of over-familiarization and boredom [66], participants were advised against consuming the test-meals daily or multiple times within the same day. As general guidance, participants were provided with a suggested consumption schedule, where one test-meal was to be consumed every 2 to 3 days until all 12 exposures were completed. Further, participants were encouraged to adapt this schedule to fit their lifestyle and maintain a consistent routine throughout the intervention. The participants were guided to consume the legume test-meals alone and with water as the only accompanying beverage. However, the participants were free to consume other foods before and after the test-meal according to their usual eating habits.

The number of exposures was based on research suggesting that 10 exposures could be sufficient to increase liking of a novel food [42]. Additional exposures were added to ensure that the minimum number of exposures would be met. The intervention period of 3 weeks was based on the findings that interpersonal variations in habit formation may take anything between 18–254 days [67].

Post-intervention: The post-intervention measurements were conducted approximately 3 weeks after the baseline. The procedure and measurements mimicked the baseline measurements, except for participants not answering sections 1–2 in the questionnaire (refer to Section 2.5).

### 2.4. Types of Stimuli

#### 2.4.1. Baseline and Post-Intervention Stimuli

The stimuli at baseline and post-intervention comprised a test stimulus of 80 g canned fava beans (La Doria S.p.A, Italy, Salling Group, Brabrand, DK; app. 50 g after cooking) and a control stimulus of 80 g butter beans (La Doria S.p.A, Italy, Salling Group, Brabrand, DK; app. 50 g after cooking). Fava beans and butter beans were selected due to their comparable size, density, and culinary characteristics, minimizing variation attributable to the food products’ physical properties. Moreover, fava beans were chosen as the target legume based on lack of familiarity with them within the Danish food context, lessening the potential influence of pre-existing flavor associations. This was a prerequisite for the theoretical foundation of the study, as Mere-Exposure, Flavor–Flavor and Flavor–Nutrient learning theory posits that the target food must be sufficiently novel or unfamiliar for any effects to occur. The control stimulus was included to investigate potential transfer effects and examine if any results discovered were distinctly related to the target legume. The portion size of 80 g was chosen as this amount corresponds to one serving of fruits and greens per day [68], supporting human nutrition. Barbecue spice and rapeseed oil were chosen due to their high level of familiarity, acceptability and widespread household use in Denmark. Their role as conditioning stimuli was intended to reflect culinary practices commonly applied in the Danish food context. Additionally, legumes generally possess a mild flavor profile and high flavor-binding capacity, making them suitable carriers to examine sensory conditioning.

Preparation of the legumes followed a standardized procedure: canned legumes were drained from brine, rinsed in tap water, dried, pan-heated for 15 min at low to medium heat, and cooled to room temperature a maximum of 20 min prior to serving. The legume test-meals were weighed and potential irregular legumes removed. A quantity of 100 mL of tap water was served alongside both the test and control stimuli as required intake.

#### 2.4.2. Mere-Exposure Stimuli

Participants in the Mere-Exposure group were instructed to prepare the fava beans in accordance with the standardized preparation method at baseline and post-intervention. As such, participants in this group consumed the fava beans pan-heated at low to medium heat, neutrally (without additional seasoning nor oil) for a total of 12 exposures at home. The participants in the Mere-Exposure group received similar test-meals at baseline, during the intervention period, and post-intervention.

#### 2.4.3. Flavor–Flavor Stimuli

Participants in the Flavor–Flavor-learning group were instructed to prepare the fava beans in accordance with the standardized preparation method at baseline and post-intervention and to add one teaspoon of barbecue spices (dried and bottled Santa Maria Barbecue Spice). Barbecue spice was used as the unconditioned stimulus, and fava beans as the conditioned stimulus. All participants in the Flavor–Flavor group were screened for a high barbecue spice preference prior to participation, and only participants with a preference of ≥8 on a 10-point scale were considered for this group. The participants in this group consumed the fava beans pan-heated, flavor-enriched for a total of 12 exposures at home. The participants received the test-meals neutrally at baseline and post-intervention, and with flavor enhancements only during the intervention period at home. Importantly, the Flavor–Flavor stimulus was removed from the post-intervention measurement test-meal to examine if the flavor exposure during the intervention period had been successfully associated with the target legume.

#### 2.4.4. Flavor–Nutrient Stimuli

Participants in the Flavor–Nutrient group were likewise instructed to prepare the fava beans in accordance with the standardized preparation method at baseline and post-intervention and to add one tablespoon of rapeseed oil (La Doria S.p.A, Italy, Salling Group DK), while pan frying the fava beans, resulting in an approx. 500 kJ addition to the meal. Rapeseed oil was used as the unconditioned stimulus, and fava beans as the conditioned stimulus. The participants in this group consumed the fava beans pan-heated, nutrient-enriched for a total of 12 exposures at home. The participants in the Flavor–Nutrient learning group received the test-meals neutrally at baseline and post-intervention, and with nutrient enrichment only during the intervention period at home. Importantly, the Flavor–Nutrient stimulus was removed from the post-intervention measurement test-meal to examine if the enriched nutrient exposure during the intervention period had been successfully associated with the target legume.

### 2.5. Questionnaire Measures

The baseline questionnaire included the following: section (1) Basic measures: demographics, attitudes towards sustainable eating behavior, eating habits, factors affecting food choice, and food sources; section (2) Attitudes towards legumes, and current consumption; section (3) Pre-consumption measures: flavor cravings, wanting to eat food, fullness prior, hunger prior, liking of the smell and appearance of the test-meal, and wanting to eat the test-meal; section (4) During consumption measures: liking of the test-meal, liking of the test-meals’ flavor and texture; section (5) Post-consumption measures: overall satisfaction of the test-meal, fullness post, hunger post, physical and mental well-being, craving more of the test-meal. Sections 3–5 were repeated for both the fava bean (test stimulus) and the butter bean (control stimulus) test-meals.

In terms of the post-intervention questionnaire, Sections 3–5 were repeated along with the test-meals being served in the exact same order. Moreover, the post-intervention questionnaire included two additional sections, namely: section (6) compliance measures: number of test-meals consumed at home, and time of consumption; and section (7) future motivation to purchase and consume legumes. All questions and reply scales are available in Supplementary Materials S1.

The first part of the questionnaire included demographic questions about age, gender, residence, number of people and children in the household, educational level, diet type and the participants’ attitudes towards sustainable eating, including the participants’ stage of change towards sustainable eating behavior. To measure the participants’ stage of change, a modified version of the Transtheoretical Model of Change [69] was applied. The model comprises six stages of change: Precontemplation, Contemplation, Preparation, Action, Maintenance and Termination/Relapse, which were reflected in the answer formats in the questionnaire. Perceived importance of a sustainable diet was measured by “How important is it to you that your food is climate friendly?” on a 10 cm line scale (1: Not important at all to 10: Extremely important). Furthermore, the first section included questions about factors that affected the participants’ food choices and eating pleasure, based on the Food Choice Questionnaire [70] and the Food Pleasure Scale [24].

The second part included a measure of overall liking of legumes on a 9-point hedonic scale (1: Do not like at all, 5: Neither dislike nor like, 9: Like very much). The participants’ current consumption of legumes was measured categorically: Never, Less than once a week, Once a week, 2–3 times a week, 4–6 times a week, or Daily.

The five appetite variables: wanting to eat food, hunger prior, fullness prior, hunger post and fullness post were measured before and after the test-meals on a 10 cm line scale (1: Not at all to 10: Very much). The well-being variables of mental and physical well-being were measured on a 10 cm line scale (1: Not at all to 10: Very much) post-consumption of the test-meals. Liking of the sensory attributes of the legumes: Smell, Appearance, Taste and Texture were similarly measured on a 10 cm line scale (1: Not at all to 10: Very much). Regarding the preference measures: Liking was measured after the first bite of the test-meals on a 9-point hedonic scale (1: Do not like at all, 5: Neither dislike nor like, 9: Like very much), while Wanting to eat the test-meal, Overall satisfaction and Craving more of the test-meal were measured on a 10 cm line scale (1: Not important at all to 10: Extremely important).

The baseline and post-intervention assessments also included questions specifically developed for the present study based on the scientific literature and behavioral theory. The questionnaire was pilot-tested and reviewed prior to commencing the study. The original questionnaire was developed and distributed in Danish but was translated for the purpose of the scientific paper.

### 2.6. Statistical Analyses

All variables were analyzed using descriptive statistics via Microsoft Excel^®^ (Microsoft, 2406, Washington, DC, USA). Subsequent statistical analyses were completed utilizing using XLSTAT^®^ (version 2024.2.2, Addison, New York, NY, USA), with a significance level set at α < 0.05 for all calculations.

#### 2.6.1. Demographic Variables and Sustainability Attitudes

Categorical variables were analyzed between groups using the Chi-square test of independence to compare group distributions, or Fisher’s exact tests, when counts were below five. The categorical variables included the following: Gender, Education, Diet type, Current legume consumption and Stage of change towards sustainable eating behavior. Continuous variables were analyzed between groups using Kruskal–Wallis tests. Dunn’s procedure was applied for post hoc multiple pairwise comparisons. Continuous variables included the following: Age, Number of people in the household, Number of children in the household, Importance of a sustainable diet and Overall legume liking.

#### 2.6.2. Appetite and Well-Being Variables

Within-group comparisons of appetite and well-being variables were conducted using Wilcoxon signed-rank tests to check potential differences prior to and post eating the test-meals at baseline and post-intervention. For the between-group comparisons, at baseline and post-intervention measurements, respectively, one-way ANOVA or Kruskal–Wallis tests were applied depending on the normality of the sample. Normality was tested using Shapiro–Wilk tests. Dunn’s procedure was applied as post hoc multiple pairwise comparisons to assess potential differences between groups. Appetite variables included the following: Wanting to eat food, Hunger prior, Hunger post, Fullness prior, Fullness post, while well-being variables included the following: Physical well-being and Mental well-being.

#### 2.6.3. Liking of Sensory Properties

The sensory variables included were as follows: Appearance, Smell, Taste and Texture. Within-group analyses were conducted using Wilcoxon signed-rank tests, while between-group comparisons were computed using Kruskal–Wallis tests and, subsequently, Dunn’s procedure post hoc testing.

#### 2.6.4. Legume Preference Variables (Fava Beans and Butter Beans)

The preference response variables—Wanting to eat the test-meal, Liking, Overall satisfaction and Craving more of the test stimuli and control stimuli—were measured at baseline and post-intervention. For within-group comparisons (i.e., within the Mere-Exposure, Flavor–Flavor and Flavor–Nutrient groups) Wilcoxon signed-rank tests were applied. Subsequently, Delta variables were calculated to examine the level of change in legume preference for all four preference variables. The Delta values (ΔPreference = Preference_Post-intervention_ − Preference_Baseline_) were analyzed using Kruskal–Wallis tests between groups. Similarly to the above, Dunn’s procedure was applied as a post hoc test when relevant. As age was found to be significantly different between groups, Pearson’s correlation tests were applied as a follow-up to test for potential correlation between age and legume preference, both within and between groups, and on Delta values. Potential carry-over effects were investigated using a secondary legume, butter beans, as a control stimulus. For within-group comparisons Wilcoxon signed-rank tests were applied, whereas between-group comparisons were conducted using Kruskal–Wallis tests and, subsequently, Dunn’s procedure post hoc testing.

## 3. Results

### 3.1. Appetite and Well-Being

No differences were found between the groups for any of the appetite and well-being measurements at baseline nor post-intervention (Table 2), with a similar level of appetite and well-being before consumption of the test-meals observed. Overall, the results showed that participants had moderate appetite and well-being levels. As an example, the participants rated ‘Wanting to eat food’ with a mean of 6.27 ± 2.02 for the Mere-Exposure group, 6.00 ± 1.91 for the Flavor–Flavor group, and 7.04 ± 1.60 for the Flavor–Nutrient group at baseline (NS).

### 3.2. Preference for the Test Stimulus (Fava Bean)

**Figure 2:**
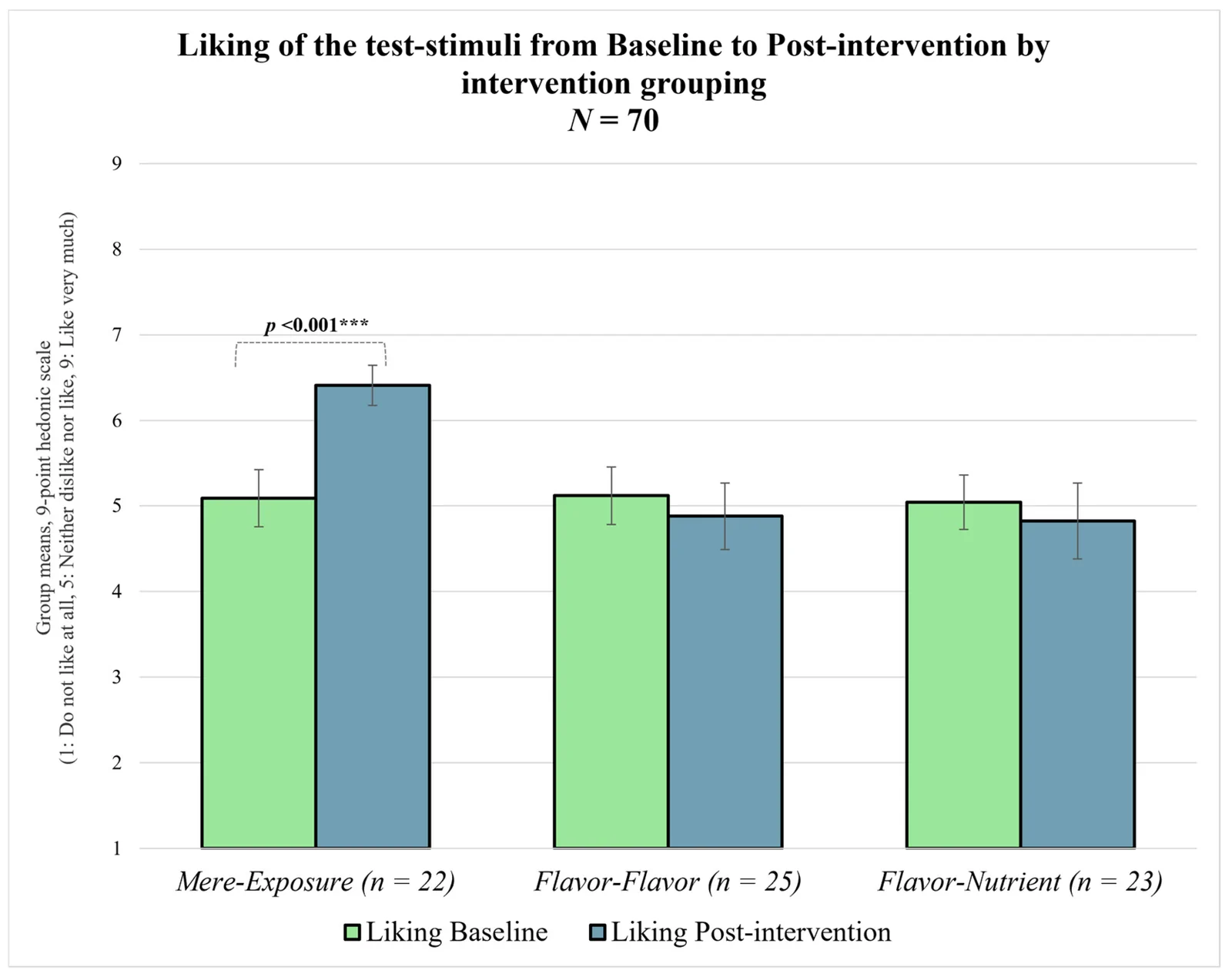
Liking of the test stimulus from baseline to post-intervention, all groups (*N* = 70). The test-stimulus consisted of fava beans, neutral. Liking within the groups from baseline to post-intervention. Significant differences were observed for the Mere-Exposure group (*n* = 22) in Liking at baseline compared to post-intervention (*p* < 0.001). No differences were observed for the Flavor–Flavor (*n* = 25) nor the Flavor–Nutrient group (*n* = 23). SEM values as error bars. * Indicates significance levels: *p* < 0.001 ***.

Preference variables included the primary factor, Liking, and secondarily, Wanting to eat the test-meal, Overall satisfaction and Wanting more of the test-meal. Significant differences in preference for the test stimulus (fava beans) were found within the Mere-Exposure group between the baseline and post-intervention measurements. For this group, Liking increased from 5.09 ± 1.57 to 6.41 ± 1.9 (*p* < 0.001), whereas no changes in Liking were observed for the Flavor–Flavor and Flavor–Nutrient groups (Figure 2). Delta values (Δ = Post − Baseline) were calculated for changes in preferences between the baseline and post-intervention measurements and analyzed for between-group differences using Kruskal–Wallis tests. Significant differences in preferences between groups were found for the variable Liking. Dunn’s procedure pairwise comparisons clarified that the Mere-Exposure (Δ: 1.32 ± 1.55) group had a higher increase in Liking compared to the Flavor–Flavor (Δ: −0.24 ± 1.82) and Flavor–Nutrient (Δ: −0.22 ± 2.59) groups, overall: DF = 2, *p* = 0.01, K = 8.70. No differences were found for the preference variables Wanting to eat the test-meal, Overall satisfaction and Craving more of the test-meal for the between-group Delta values (not illustrated).

**Figure 3:**
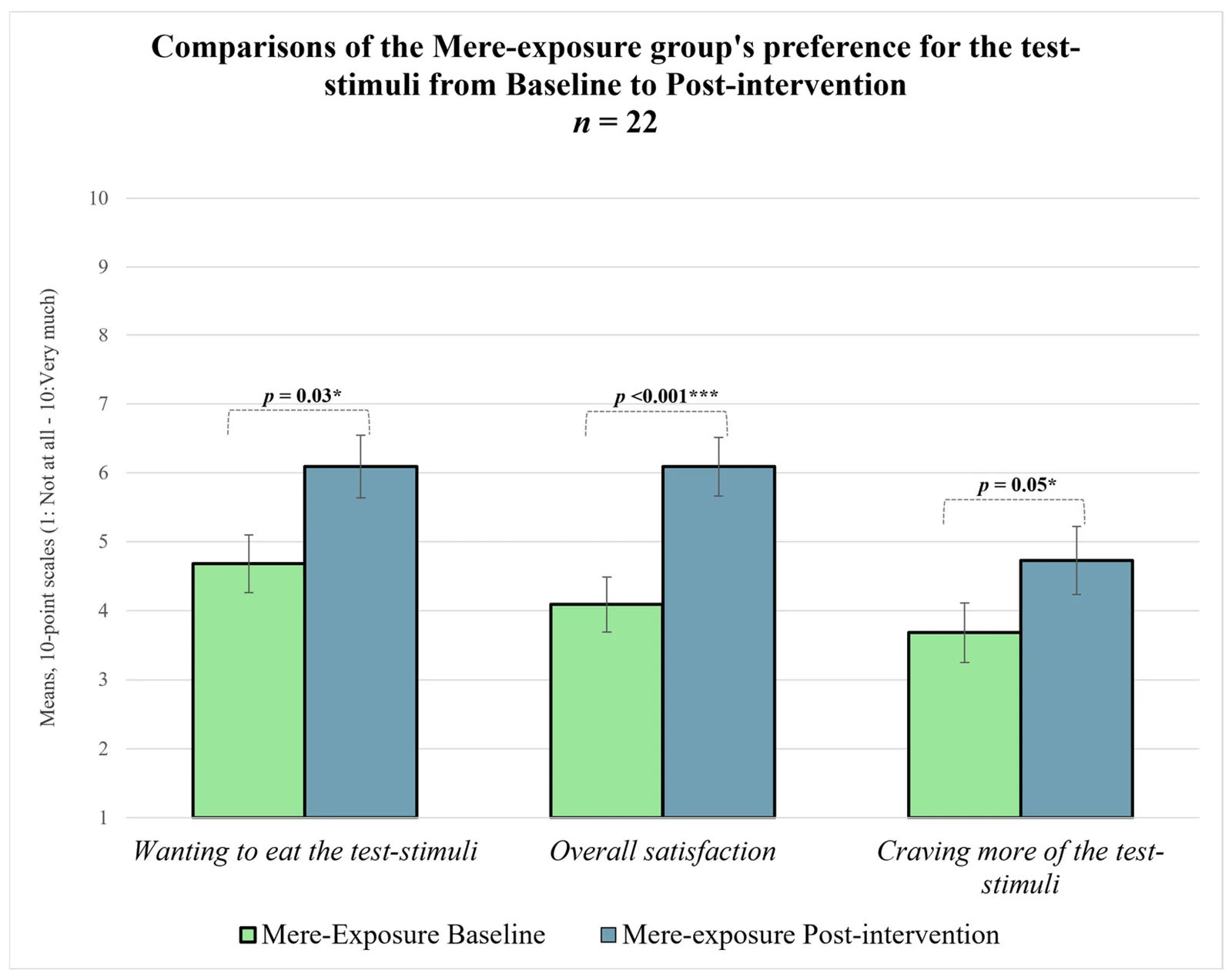
Comparison of the Mere-Exposure group’s preference for the test stimulus from baseline to post-intervention. The test stimulus consisted of fava beans, neutral. Mean values on 10 cm scales (1: Not at all to 10: Very much). Significant differences were found for the following: Wanting to eat the test stimulus: *p* = 0.03, Overall satisfaction: *p* < 0.001 and Craving more of the test-meal: *p* = 0.05. SEM values as error bars. * Indicates significance levels: *p* < 0.05 *, *p* < 0.001 ***.

**Figure 4:**
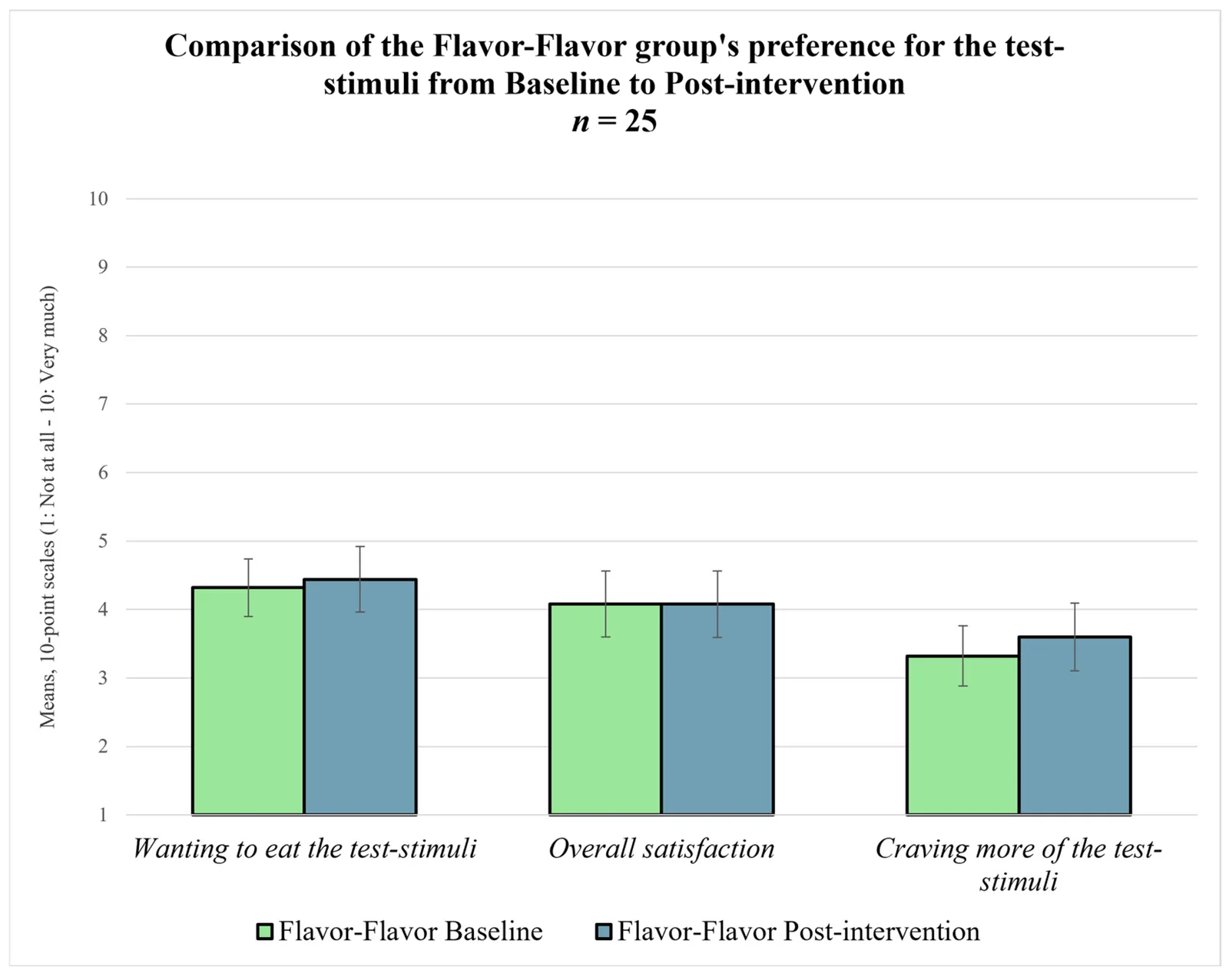
Comparison of the Flavor–Flavor group’s preference for the test stimulus from baseline to post-intervention. The test stimulus consisted of fava beans, neutral. Mean values on 10 cm scales (1: Not at all to 10: Very much). No differences were observed. SEM values as error bars.

**Figure 5:**
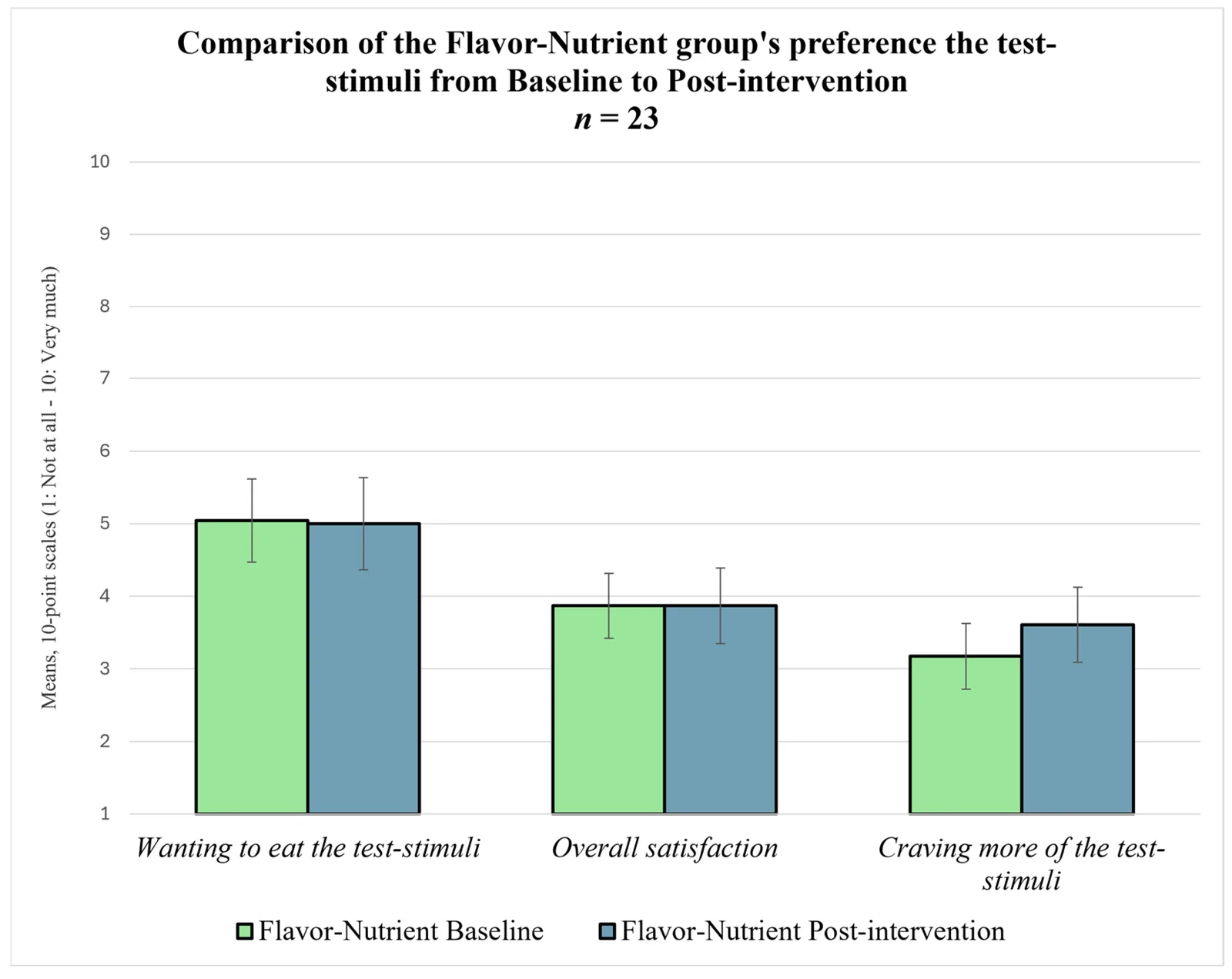
Comparison of the Flavor–Nutrient group’s preference for the test stimulus from baseline to post-intervention. The test stimulus consisted of fava beans, neutral. Mean values on 10 cm scales (1: Not at all to 10: Very much). No differences were observed. SEM values as error bars.

In conjunction with the increased Liking for fava beans within the Mere-Exposure group, Wanting to eat the test-meal increased significantly from a mean of 4.68 ± 1.96 at baseline to 6.09 ± 2.13 post-intervention (*p* = 0.03), Overall satisfaction increased from 4.09 ± 1.87 to 6.09 ± 1.99 (*p* < 0.001), and Craving more of the test-meal increased from 3.68 ± 2.03 at baseline to 4.73 ± 2.31 post-intervention (*p* = 0.05) (see Figure 3). No changes were found for Wanting to eat the test-meal, Overall Satisfaction and Craving for more within the Flavor–Flavor (Figure 4) nor Flavor–Nutrient (Figure 5) groups when comparing their respective baseline and post-intervention measurements.

### 3.3. Liking of the Test Stimulus’ Sensory Properties (Fava Bean)

Significant differences were found within the Mere-Exposure group’s ratings of liking of sensory properties from baseline to post-intervention. The participants displayed an increase in mean liking of the test stimulus’ Appearance from baseline 3.18 ± 1.84 to 4.36 ± 1.73 (*p* = 0.02) post-intervention, and Flavor from baseline 5.00 ± 1.54 to 6.27 ± 1.88 (*p* = 0.01) post-intervention. No differences were found for liking of Smell or Texture in the Mere-Exposure group (Table 3). For the Flavor–Nutrient group, a significant difference was found for liking of Appearance from a baseline mean of 2.38 ± 1.66 to 3.91 ± 2.08 at post-intervention (*p* = 0.04), whereas no difference was found for any of the variables focusing on liking of sensory properties within the Flavor–Flavor group.

### 3.4. Preference for the Control Stimulus (Butter Bean)

The participants’ preference for a secondary legume, butter beans, was measured and compared to investigate potential transfer effects of the intervention. Mean Liking for the control stimulus within the intervention groups was as follows: Mere-Exposure baseline 4.63 ± 1.71 to 4.90 ± 1.89 post-intervention, Flavor–Flavor baseline 3.96 ± 1.98 to 4.12 ± 2.06 post-intervention, and Flavor–Nutrient baseline 4.47 ±1.99 to 4.21 ± 1.85 post-intervention. Kruskal–Wallis analyses revealed no significant differences between baseline and post-intervention ratings of Liking within the Mere-Exposure (Δ: 0.27 ± 1.68), Flavor–Flavor (Δ: 0.16 ± 1.31) nor Flavor–Nutrient (Δ: −0.26 ± 2.43) groups, and neither were any difference in Delta Liking values (Δ_Liking_ = Post_Liking_ − Baseline_Liking_) found between the three groups. Thus, comparisons of the three sensory learning strategies showed no transfer effect from the target legumes, fava beans, to the control legume, butter beans.

## 4. Discussion

The study aimed to compare the effects of Mere-Exposure, Flavor–Flavor and Flavor–Nutrient learning strategies on adult consumers’ preference for an unfamiliar legume, fava beans. Based on the previous literature, the Mere-Exposure strategy was expected to result in the highest increase in preference, compared to the Flavor–Flavor and Flavor–Nutrient learning strategies. This hypothesis was confirmed in the present study, where the Mere-Exposure strategy was found to be the most effective. Still, the latter strategies were included as they represent well-established sensory conditioned learning strategies. The present study adds a comparison of the effectiveness of the strategies in a Danish adult sample to the literature and discussion of whether any of the strategies were shown to increase legume preferences. Overall, it should be considered that consumer preferences can evolve and thus interventions aimed at shifting consumer preferences can provide guidance for further studies.

### 4.1. Comparing Mere-Exposure, Flavor–Flavor and Flavor–Nutrient Learning in Increasing Preference for a Legume

Comparing all three sensory learning strategies, it was found that only the participants following the Mere-Exposure strategy increased their preference for the neutral fava beans after 12 exposures. The study found no supporting evidence for an effect of the Flavor–Flavor nor Flavor–Nutrient learning strategies on preference for neutral fava beans. Other comparisons of Mere-Exposure, Flavor–Flavor and Flavor–Nutrient learning similarly underscore the findings of Mere-Exposure as being the most effective at increasing preferences and acceptance of an unfamiliar food [39,41,42,43,47]. However, previous studies have mostly been conducted in children or adolescents, where preferences are more easily modified, due to still being at developmental stages and the effects of sensory conditioned learning in adults are unclear. As other studies have argued, learning generally takes place in so-called age-related developmental windows [32,71,72]. Since the mean age of the Mere-Exposure group was lower than the mean age of the Flavor–Flavor and Flavor–Nutrient groups, it could be hypothesized that the developmental window, in which associative learning takes place, was passed for the participants in the Flavor–Flavor and Flavor–Nutrient groups. Follow-up Pearson’s correlation tests were conducted on Delta overall Liking values (from baseline to post-intervention) to examine a potential correlation between age and differences in Liking. No significant correlation (*r* = 0.15, NS) was found for the Mere-Exposure group, indicating that the younger age was not a significant driver of a larger increase in Liking within this group and vice versa. However, a significant negative correlation was found within the Flavor–Flavor (*r* = −0.48, *p* = 0.01) and Flavor–Nutrient groups (*r* = −0.44, *p* = 0.03), indicating that the higher age in these groups could drive the lower Liking ratings in these groups. Notably, not much is known currently about shifting consumers’ food preferences at a higher age, whereby the present study contributes with meaningful insights. It should be considered that the results found in the present study are more preliminary than conclusive and further research is needed within adult samples to confirm the effects of sensory conditioning at later stages in life.

Sensory conditioning studies have suggested that for learning strategies to be effective, specific circumstances must also be present, for example, the target food used must be sufficiently novel and initially neutrally liked or disliked [52,73,74]. The participants were screened for their familiarity with fava beans, and Liking for the test stimulus (fava beans) was shown to be neutral at baseline (Mere-Exposure mean: 5.09 ± 1.57, Flavor-Flavor mean: 5.12 ± 1.69, Flavor-Nutrient mean: 5.04 ± 1.52, measured on a 10-point scale). From this, it can be deduced that pre-existing familiarity or preference could not be the main explanatory factor for the lack of effect of the Flavor–Flavor and Flavor-Nutrient learning strategies.

In terms of Flavor–Flavor learning, associative learning theory argues that the pairing of a neutral or disliked flavor with a positive unconditioned stimulus will result in a positive shift in liking of the initially neutral or disliked flavor [36,54,74]. The unconditioned stimulus must be sufficiently liked (i.e., the barbecue spice in the current study) for the conditioned stimulus (i.e., the fava bean) to demonstrate a shift in the direction of the unconditioned stimulus. Additionally, it is important that the conditioned stimulus (i.e., the fava bean) is neutral or disliked before a form of Pavlovian conditioning can occur [35,36,54]. Upon enrollment in the study, all participants were screened for a high preference for barbecue spice, meaning the barbecue spice can be argued as being an appropriate unconditioned stimulus. Moreover, the participants had neutral liking of fava beans at baseline (approx. 5 on a 10-point scale), meaning the underlying conditions were in place for the strategy to show an effect. As the theoretical foundation for the sensory conditioning to succeed was present in the current study, the lack of detectable effects may be related to other challenges associated with modifying pre-existing flavor preferences in an adult sample. In this case, food preferences are often well established and habitualized, and legumes may be viewed as less tasty [58], which was also reflected in the Flavor–Flavor groups’ responses to the sensory evaluation of the fava beans post-intervention. As a final remark, the lack of results could also relate to the participants’ evaluation of the barbecue spice and that the spice was not a suitable component to pair with fava beans. This evaluation was nevertheless not tested amongst the participants at the post-intervention measurements, and therefore that perspective is purely speculative.

Another circumstance relates to the Flavor–Nutrient strategy and argues that for changes in liking to take place via positive post-ingestive associations, participants need to be in a state of hunger, as hunger and fullness modifies liking perceptions [75]. From the results presented in Section 3.1, it can be observed that the Flavor–Nutrient learning group reported a mean fullness rating below neutral, and a hunger rating around neutral at baseline, indicating that this circumstance was fulfilled on-site and could not be the reason for the strategy not showing an effect. Yet, due to the natural setting of the intervention period, it cannot be guaranteed that this condition (i.e., being in a state of hunger) was fulfilled during the 12 exposures to unconditioned stimuli in the Flavor–Nutrient group. The results should be carefully interpreted within this context. The rapeseed oil dose provided approximately 500 kJ, which may have been insufficient to elicit a detectable post-ingestive signal in adult participants. A similar lack of effects has been observed in other studies [76] where the participants did not ingest enough of the test stimuli to result in any noticeable post-ingestive effects. It is likewise possible that the participants ingested other foods following the test stimulus, which could have influenced the post-ingestive sensations they experienced.

From a theoretical standpoint, the success of Flavor–Flavor and Flavor–Nutrient learning relies on the formation of associations, where the hedonic value of a well-liked flavor or nutrient enrichment must be transferred to the neutrally liked, unfamiliar legume. The absence of an increase in preference suggests a failure in associative transfer, indicating that either the chosen unconditioned stimuli were insufficient to override the pre-existing preferences of fava beans, or it could be that adult reward pathways are less receptive. From another standpoint, the lack of increase in preference from the Flavor–Flavor and Flavor–Nutrient learning groups points towards the suggestion that the results found for the Mere-Exposure group cannot be attributed to random variation alone. If that was the case, an increase in preference might have been observed across all groups. Our findings add to the literature showing that sensory conditioning in humans is rather complex. This interpretation is further supported by Yeomans [48], who argued that while Flavor–Flavor learning mechanisms can operate in humans, their effects appear harder to detect compared to animal studies. The mechanism behind Flavor–Flavor and Flavor–Nutrient learning may therefore be more to do with secondary mechanisms of identifying safe and nutritious foods. Subsequently, Yeomans [48] indicated that other forms of learning, including social influences and expectations, could play a more substantial role in shaping food preferences. To date, the conditions in which especially Flavor–Flavor and Flavor–Nutrient learning can be optimally demonstrated have not yet been detailed [48,76,77].

Lastly, the specific stimulus chosen could have had an influence on the study results. While the use of barbecue spice and rapeseed oil is common practice in the Danish food context, the combination of the sensory conditioning stimuli with fava beans should be considered novel. The stimulus was chosen to pair an unfamiliar legume with flavors and nutrient-dense food sources familiar to Danish consumers, i.e., barbecue spice and rapeseed oil. As fava beans constitute an emerging food source in Denmark, the study participants were deemed unlikely to have strong pre-existing associations with the product, which was a prerequisite of the theoretical foundations of the study. Nevertheless, familiarity with specific flavor–food combinations may influence the effect of the associative learning processes, and therefore, alternative pairings that more-closely align with common culinary practices could be explored in future research. At the same time, the novelty of the legume and the flavor-binding properties of fava beans make them appropriate for testing conditioning approaches aimed at increasing the acceptance of sustainable protein sources for the benefit of human health and the environment.

### 4.2. Sensory Perceptions

In the context of legumes, inferior sensory perceptions are often reported as barriers to consumption [19,21,26]. Analyses of the liking of sensory properties in the current study revealed that the increase in preference for fava beans in the Mere-Exposure group occurred alongside a significant improvement in liking of Appearance and Flavor. While prior research has primarily focused on overall liking outcomes or an increase in consumption [40,42,47], these results indicate that Mere-Exposure may also operate at the level of specific sensory modalities, which represents an underexplored field of learning in adult samples. As Danes are often challenged by inferior sensory perceptions of legumes, it is particularly noteworthy that the Mere-Exposure strategy seemingly increased the participants’ liking of the flavor of fava beans. Sensory pleasure has been identified as a key driver of food choice and eating behavior, making enhanced flavor liking an important pathway through which repeated exposure may support future legume acceptance [24,53].

Another barrier to legumes is the distaste for the textural aspects of the food [65,78]. Preceding studies have found that when the texture of food aligns with an individual’s preferences, it may elicit hedonic responses and enhance texture perceptions [79,80]. On the contrary, food texture is also a common cause for food rejection [81]. As no effect on liking of Texture was found in the present study, it can be suggested that Texture represents a more challenging sensory modality to alter preference for, compared to Appearance and Flavor. While the results show overall promise, it is suggested that future studies focus on the improvement of sensory modalities as a result of sensory conditioning strategies to investigate the holistic effects on increasing adult preferences for more sustainable foods.

### 4.3. Transfer Effects

A secondary aim of the study was to explore potential transfer effects to a different legume (butter beans), serving as an internal control stimulus. Exposure effects may be transferred to other, unexposed stimuli, which are similar to the exposed target stimuli [82], where the effects can extend beyond the included target legume (fava beans). Evidence for transfer effects remains unclear and is especially underexplored in adults. Sullivan et al. [83] found no transfer effects in preschool children repeatedly exposed to novel tofu preparations with salt and sugar added or to similar foods such as ricotta and Jicama, whereas Hausner et al. [42] found transfer effects following exposure to a novel artichoke purée in 2–3-year-old children, with carrot purée as a control stimulus. Birch [84] further discusses associative food preference development in children, stating that most learning occurs through experiences with food and eating, or the eating environment, social and physiological factors. As the existing evidence is primarily based on children or younger populations, it remains unknown whether comparable transfer effects can occur in adults, whose food preferences generally tend to be more well established.

In the current study, the increases in preference reported in the Mere-Exposure group could potentially extend beyond preference for fava beans to also increase preference for butter beans, if the stimuli were sufficiently similar. However, as the results showed no changes in Liking for butter beans from baseline to post-intervention, no transfer effects could be concluded. Though both stimuli are within the category of legumes, which resemble each other in sensory characteristics compared to foods from other categories, it is possible that the sensory properties of the two stimuli were too distinct for transfer effects to occur. In the study by Hausner and colleagues [42], resemblance in texture properties specifically seemed to drive the transfer effects. As no Mere-Exposure effect on liking for the Texture of fava beans was found in our study, and Danes often express aversion to the texture of legumes [65], these circumstances may have limited the likelihood of transfer effects even further.

### 4.4. Strengths and Limitations of the Study

To the authors’ knowledge, no previous studies have compared the potential effects of Mere-Exposure, Flavor–Flavor and Flavor–Nutrient learning in an adult sample with a focus on sustainable eating behavior. This study, therefore, offers a unique insight into the applicability of sensory conditioned learning strategies and their effect on adult legume preferences. Additionally, the current study investigated the effect of the three interventions strategies on key sensory modalities, which has received limited attention in previous sensory conditioning studies. Another strength of the study is that it was conducted in the participants’ natural settings. Conducting an intervention study in a natural setting increases ecological validity and captures more genuine behavioral responses within real-world variables, which are difficult to duplicate in laboratory conditions.

Conducting the study in the participants’ natural settings created both opportunities and limitations. Due to the ecological validity of the study during the intervention period, variability in the preparation of the food samples might have occurred. Nevertheless, as each participant received both verbal and written instructions outlining the exact preparation method of the fava beans in line with her/his exposure group, this risk of not preparing the fava beans according to the learning strategy seems minimal. Furthermore, a slight gender imbalance within the exposure groups was present, with two-thirds of the groups consisting of women compared to men. In general, women are known to express more positive responses towards plant-based foods [85], but as the gender distribution was equal in all groups, this was unlikely to have affected the results in a major way. While some additional baseline characteristics (Table 1), such as Stage of change (*p* = 0.09) and Consumption frequency (*p* = 0.42), appeared numerically uneven across intervention groups, Chi-square and Fisher’s exact statistical comparisons indicated no significant differences between groups. The three intervention groups were therefore deemed appropriate prior to further analyses. Although the sample size was comparable to those reported in previous sensory-learning intervention studies [39], and was sufficient to detect medium-to-large effects according to the sensitivity analysis, future studies may benefit from a larger sample to more adequately detect smaller effects. The study also employed a within-subject comparison of Mere-Exposure, Flavor–Flavor and Flavor–Nutrient learning, whereby a ‘no strategy’ group was not included. The within-subject design was chosen as a suitable design for comparing the three sensory learning conditions, as this would cancel out natural variability between participants. It is acknowledged that a control group could bolster future studies to investigate whether the results obtained were specific to this consumer group. Furthermore, as overall liking and attribute-specific measures were assessed using different answer formats, direct comparison of absolute results should be interpreted within this context, and alternative measures may be explored in future research.

Future studies could also benefit from the inclusion of longitudinal measures of habit and habit strength, i.e., through repeated dietary tracking or validated habit formation scales, to validate the long-term effects of sensory conditioned learning strategies on eating behavior. Finally, the inclusion of cognitive and affective measures, such as of emotions and memories, in further research is also recommended, as the aforementioned factors play a key role in preference formation.

## 5. Conclusions

The Mere-Exposure strategy showed promise in increasing adult consumers’ preference for a target legume. Furthermore, the Mere-Exposure strategy fostered an increased liking of the test stimuli’s Appearance and Flavor, whereas the Flavor–Flavor- and Flavor–Nutrient learning groups exhibited no effect on legume preference. No evidence of transfer effects was observed to a secondary legume, as the effect of Mere-Exposure on increasing preference did not generalize to a control stimulus. Based on our comparisons of the strategies, Mere-Exposure was found to be the most promising strategy under the conditions of the present study. In the context of the transformation of our global food systems, enhancing preference for even a single legume variety represents potential for future developments. Given that adult food preferences are often resistant to change, studies that focus on overcoming sensory-related issues to legume consumption can serve as the first step towards integrating more sustainable, plant-based proteins in adult diets, contributing to broader health and climate mitigation goals.

## Figures and Tables

**Table 1:** Participant characteristics for the exposure groups. No difference between groups was found for any of the sociodemographic characteristics except age (*p* = 0.01). The Mere-Exposure group had a lower mean age, compared to the Flavor–Flavor and Flavor–Nutrient groups. The latter part of the table includes the attitudinal responses to sustainable eating behavior for each group. No differences in overall liking of legumes between the groups prior to the intervention were present. Not significant (NS).

	Mere-Exposure	Flavor–Flavor	Flavor–Nutrient	*p*-Value
Number of participants (Men/Women/Other)	22 (8/13/1)	25 (9/16/0)	23 (7/14/2)	NS
Age, range (median)	19–41 (24.50)	22–73 (30)	19–81 (28)	*p* = 0.01
Education				NS
Primary school/Lower secondary school	0%	0%	9%	
High school	41%	20%	26%	
Vocational education	0%	4%	4%	
Short higher education, 2 years	0%	8%	9%	
Medium-length higher education, 3–4 years	41%	48%	35%	
Long higher education, +5 years	18%	20%	17%	
Diet				NS
Omnivore	45%	60%	48%	
Flexitarian	36%	36%	39%	
Pescetarian	14%	0%	9%	
Vegetarian	5%	4%	4%	
Number of people in the household, range (median)	1–10 (2)	1–13 (2)	1–9 (2)	NS
Number of children under 18 in the household, range (median)	0–1 (0)	0–3 (0)	0–4 (0)	NS
Stage of change, %				NS
(1) I have no intention of behavior change.	5%	16%	9%	
(2) I am aware of the necessary behavior change but have yet to commit myself to change to a more sustainable eating behavior.	5%	24%	35%	
(3) I am beginning to plan to make changes to a more sustainable eating behavior and feel obligated to follow through.	50%	24%	17%	
(4) I have implemented the desired behavior changes, in an attempt to change to a more sustainable eating behavior.	14%	20%	17%	
(5) I am maintaining a more- sustainable eating behavior and try to avoid going back to a less-sustainable eating behavior.	27%	16%	13%	
(6) I have not been able to maintain a more-sustainable eating behavior.	0%	0%	9%	
Importance of a sustainable diet, mean	6.77 ± 1.60	6.84 ± 1.79	6.56 ± 2.10	NS
Current legume consumption				NS
Never	0%	4%	0%	
Less than once a week	23%	12%	17%	
Once a week	18%	28%	30%	
2–3 times a week	41%	52%	39%	
4–6 times a week	18%	0%	9%	
Daily	0%	4%	4%	
Overall liking of legumes, mean	7.0 ± 1.19	6.6 ± 1.15	6.78 ± 0.99	NS

**Table 2:** Appetite and well-being measurements prior to and after consuming the test stimuli at baseline and post-intervention. All groups (*n* = 70). Mean values ± standard deviation (Likert scale: 1: Not at all to 10: Very much). No differences were found for the between-group comparisons. Wanting to eat food, Fullness and Hunger were measured prior to consumption of the test-meals, whereas Physical well-being and Mental well-being were measured post-consumption. Not significant (NS).

	Baseline			Post-Intervention			
	Mere-Exposure	Flavor–Flavor	Flavor–Nutrient	Mere-Exposure	Flavor–Flavor	Flavor–Nutrient	Between-Group Comparisons
Wanting to eat food	6.27 ± 2.02	6.00 ± 1.91	7.04 ± 1.60	6.18 ± 1.76	5.64 ± 2.09	6.39 ± 2.08	NS
Fullness	3.32 ± 2.07	3.48 ± 1.98	3.57 ± 1.64	3.18 ± 1.36	3.36 ± 1.95	3.96 ± 1.63	NS
Hunger	5.82 ± 2.10	5.64 ± 2.03	6.52 ± 2.06	6.73 ± 1.48	5.68 ± 2.11	6.00 ± 2.17	NS
Physical well-being	5.18 ± 2.17	4.84 ± 2.46	4.26 ± 2.33	5.91 ± 1.99	5.16 ± 2.46	4.83 ± 2.47	NS
Mental well-being	4.83 ± 1.91	4.20 ± 2.30	4.30 ± 2.60	5.77 ± 1.97	4.56 ± 2.32	4.70 ± 2.72	NS

**Table 3:** Within-group liking of sensory properties while consuming the test stimulus at baseline and post-intervention. Mere-Exposure (*n* = 22), Flavor–Flavor (*n* = 25) and Flavor–Nutrient (*n* = 23). Wilcoxon signed-rank tests within groups. Mean values (Likert scale 1: Not at all to 10: Very much). Significant increase in liking of the test stimulus’ Appearance (*p* = 0.02) and Flavor (*p* = 0.01) was found for the Mere-Exposure group, and Appearance (*p* = 0.04) for the Flavor–Nutrient group. Not significant (NS).

	Mere-Exposure			Flavor–Flavor			Flavor–Nutrient		
	Baseline	Post-Intervention	Within-Group Comparisons	Baseline	Post-Intervention	Within-Group Comparisons	Baseline	Post-Intervention	Within-Group Comparisons
Smell	4.86 ± 2.12	5.18 ± 2.21	NS	4.68 ± 1.90	4.16 ± 2.19	NS	4.48 ± 2.46	4.96 ± 2.40	NS
Appearance	3.18 ± 1.84	4.36 ± 1.73	*p* = 0.02	3.12 ± 1.61	3.44 ± 1.73	NS	2.82 ± 1.66	3.91 ± 2.08	*p* = 0.04
Flavor	5.00 ± 1.54	6.27 ± 1.88	*p* = 0.01	5.08 ± 1.97	4.8 ± 2.06	NS	5.09 ± 1.64	5.00 ± 2.39	NS
Texture	4.86 ± 1.90	5.82 ± 1.91	NS	5.08 ± 2.39	4.96 ± 2.13	NS	5.17 ± 2.28	5.04 ± 2.38	NS

## Data Availability

The datasets generated for this study are available upon request to the corresponding author.

## Supplementary Materials

The following supporting information can be downloaded at: https://www.mdpi.com/article/10.3390/foods15162884/s1, Supplementary file S1: Full questionnaire overview: schematic conceptualization of key dimensions of the baseline and post-intervention questionnaire, including the questionnaire sections, questions and answer formats.

## References

[1] Bessada S.M.F., Barreira J.C.M., Oliveira M.B.P.P. (2019). Pulses and Food Security: Dietary Protein, Digestibility, Bioactive and Functional Properties. Trends Food Sci. Technol..

[2] Mitchell D.C., Lawrence F.R., Hartman T.J., Curran J.M. (2009). Consumption of Dry Beans, Peas, and Lentils Could Improve Diet Quality in the US Population. J. Am. Diet. Assoc..

[3] Polak R., Phillips E.M., Campbell A. (2015). Legumes: Health Benefits and Culinary Approaches to Increase Intake. Clin. Diabetes.

[4] Stagnari F., Maggio A., Galieni A., Pisante M. (2017). Multiple Benefits of Legumes for Agriculture Sustainability: An Overview. Chem. Biol. Technol. Agric..

[5] Peoples M.B., Hauggaard-Nielsen H., Huguenin-Elie O., Jensen E.S., Justes E., Williams M., Lemaire G., Carvalho P.C.D.F., Kronberg S., Recous S. (2019). The Contributions of Legumes to Reducing the Environmental Risk of Agricultural Production. Agroecosystem Diversity.

[6] Trinidad T.P., Mallillin A.C., Loyola A.S., Sagum R.S., Encabo R.R. (2010). The Potential Health Benefits of Legumes as a Good Source of Dietary Fibre. Br. J. Nutr..

[7] Department of Agriculture Beans, Peas, and Lentils | MyPlate. https://myplate.food/food-groups/protein-foods.

[8] FAO International Year of Pulses 2016. https://openknowledge.fao.org/server/api/core/bitstreams/3adde3c2-79c7-4f94-81f0-75115726159c/content.

[9] Hughes J., Pearson E., Grafenauer S. (2022). Legumes—A Comprehensive Exploration of Global Food-Based Dietary Guidelines and Consumption. Nutrients.

[10] Styrelsen for Fødevarer, Landbrug og Fiskeri Mindre Frugt, Mere Slik og Lidt Flere Bælgfrugter: Ny Rapport Viser Store Skift i de Danske Madvaner. https://foedevarestyrelsen.dk/nyheder/pressemeddelelser/2026/feb/dansda.

[11] EAT-Lancet Commission The Planetary Health Diet. https://eatforum.org/eat-lancet-commission/the-planetary-health-diet-and-you/.

[12] Styrelsen for Fødevarer, Landbrug og Fiskeri Spis Mindre Kød—Vælg Bælgfrugter og Fisk. https://foedevarestyrelsen.dk/kost-og-foedevarer/alt-om-mad/de-officielle-kostraad/kostraad-til-dig/om-de-officielle-kostraad/spis-mindre-koed-vaelg-baelgfrugter-og-fisk.

[13] Mertens E., Kuijsten A., Dofková M., Mistura L., D’Addezio L., Turrini A., Dubuisson C., Favret S., Havard S., Trolle E. (2019). Geographic and Socioeconomic Diversity of Food and Nutrient Intakes: A Comparison of Four European Countries. Eur. J. Nutr..

[14] Fagt S., Christensen T., Biltoft-Jensen A., Matthiessen J., Christensen C., Rosenlund Sørensen M., Henriksen K., McClure S., Trolle E., DTU Fødevareinstituttet (2026). Main Results: Dietary Habits in Denmark 2021–2024.

[15] Foyer C.H., Lam H.-M., Nguyen H.T., Siddique K.H.M., Varshney R.K., Colmer T.D., Cowling W., Bramley H., Mori T.A., Hodgson J.M. (2016). Neglecting Legumes Has Compromised Human Health and Sustainable Food Production. Nat. Plants.

[16] Jallinoja P., Niva M., Latvala T. (2016). Future of Sustainable Eating? Examining the Potential for Expanding Bean Eating in a Meat-Eating Culture. Futures.

[17] Kuosmanen S., Niva M., Pajari A.-M., Korhonen K., Muilu T., Konttinen H. (2023). Barriers Associated with Pulse and Plant-Based Meat Alternative Consumption Across Sociodemographic Groups: A Capability, Opportunity, Motivation, Behaviour Model Approach. Front. Nutr..

[18] Onwezen M.C., Bouwman E.P., Reinders M.J., Dagevos H. (2021). A Systematic Review on Consumer Acceptance of Alternative Proteins: Pulses, Algae, Insects, Plant-Based Meat Alternatives, and Cultured Meat. Appetite.

[19] Siddiqui S.A., Alvi T., Sameen A., Khan S., Blinov A.V., Nagdalian A.A., Mehdizadeh M., Adli D.N., Onwezen M. (2022). Consumer Acceptance of Alternative Proteins: A Systematic Review of Current Alternative Protein Sources and Interventions Adapted to Increase Their Acceptability. Sustainability.

[20] Tso R., Lim A.J., Forde C.G. (2021). A Critical Appraisal of the Evidence Supporting Consumer Motivations for Alternative Proteins. Foods.

[21] Vainio A., Niva M., Jallinoja P., Latvala T. (2016). From Beef to Beans: Eating Motives and the Replacement of Animal Proteins with Plant Proteins Among Finnish Consumers. Appetite.

[22] van der Weele C., Feindt P., van der Goot A.J., van Mierlo B., van Boekel M. (2019). Meat Alternatives: An Integrative Comparison. Trends Food Sci. Technol..

[23] Jakobsen I.T., Onwezen M.C., Wang Y., Zhong F., Byrne D.V., Andersen B.V. (2026). Cross-Cultural Consumer Acceptance of Sustainable Protein-Rich Foods; Legumes, Plant-Based Meat Analogues and Hybrids, and Cell-Based Foods. Food Qual. Prefer..

[24] Andersen B.V., Chan R.C.K., Byrne D.V. (2021). A Conceptual Framework for Multi-Dimensional Measurements of Food Related Pleasure—The Food Pleasure Scale. Foods.

[25] Hyldelund N.B., Byrne D.V., Andersen B.V. (2022). Food Pleasure Profiles—An Exploratory Case Study of the Relation Between Drivers of Food Pleasure and Lifestyle and Personality Traits in a Danish Consumer Segment. Foods.

[26] Lea E., Worsley A., Crawford D. (2005). Australian Adult Consumers’ Beliefs About Plant Foods: A Qualitative Study. Health Educ. Behav..

[27] Steiner J.E. (1979). Human Facial Expressions in Response to Taste and Smell Stimulation. Adv. Child Dev. Behav..

[28] Cowart B.J., Beauchamp G.K. (1986). The Importance of Sensory Context in Young Children’s Acceptance of Salty Tastes. Child Dev..

[29] Schwartz C., Issanchou S., Nicklaus S. (2009). Developmental Changes in the Acceptance of the Five Basic Tastes in the First Year of Life. Br. J. Nutr..

[30] Bernal A., Zafra M.A., Simón M.J., Mahía J. (2023). Sodium Homeostasis, a Balance Necessary for Life. Nutrients.

[31] Fessler D.M.T. (2003). An Evolutionary Explanation of the Plasticity of Salt Preferences: Prophylaxis Against Sudden Dehydration. Med. Hypotheses.

[32] Birch L.L. (1999). Development of Food Preferences. Annu. Rev. Nutr..

[33] Karolkowski A., Belloir C., Briand L., Salles C. (2023). Non-Volatile Compounds Involved in Bitterness and Astringency of Pulses: A Review. Molecules.

[34] Spill M., Callahan E., Johns K., Shapiro M., Spahn J.M., Wong Y.P., Terry N., Benjamin-Neelon S., Birch L., Black M. (2019). Repeated Exposure to Foods and Early Food Acceptance: A Systematic Review.

[35] Rozin P., Zellner D. (1985). The Role of Pavlovian Conditioning in the Acquisition of Food Likes and Dislikes. Ann. N. Y. Acad. Sci..

[36] Baeyens F., Crombez G., Hendrickx H., Eelen P. (1995). Parameters of Human Evaluative Flavor-Flavor Conditioning. Learn. Motiv..

[37] Sclafani A. (1991). Conditioned Food Preferences. Bull. Psychon. Soc..

[38] Zajonc R.B. (1968). Attitudinal Effects of Mere Exposure. J. Pers. Soc. Psychol..

[39] Appleton K.M., Hemingway A., Rajska J., Hartwell H. (2018). Repeated Exposure and Conditioning Strategies for Increasing Vegetable Liking and Intake: Systematic Review and Meta-Analyses of the Published Literature. Am. J. Clin. Nutr..

[40] de Wild V.W.T., de Graaf C., Jager G. (2013). Effectiveness of Flavour Nutrient Learning and Mere Exposure as Mechanisms to Increase Toddler’s Intake and Preference for Green Vegetables. Appetite.

[41] Harrison A.A., Berkowitz L. (1977). Mere Exposure. Advances in Experimental Social Psychology.

[42] Hausner H., Olsen A., Møller P. (2012). Mere Exposure and Flavour–Flavour Learning Increase 2–3 Year-Old Children’s Acceptance of a Novel Vegetable. Appetite.

[43] Pliner P. (1982). The Effects of Mere Exposure on Liking for Edible Substances. Appetite.

[44] Dermiki M., Prescott J., Sargent L.J., Willway J., Gosney M.A., Methven L. (2015). Novel Flavours Paired with Glutamate Condition Increased Intake in Older Adults in the Absence of Changes in Liking. Appetite.

[45] Yeomans M.R., Mobini S., Elliman T.D., Walker H.C., Stevenson R.J. (2006). Hedonic and Sensory Characteristics of Odors Conditioned by Pairing with Tastants in Humans. J. Exp. Psychol. Anim. Behav. Process..

[46] Yeomans M.R., Leitch M., Gould N.J., Mobini S. (2008). Differential Hedonic, Sensory and Behavioral Changes Associated with Flavor–Nutrient and Flavor–Flavor Learning. Physiol. Behav..

[47] De Wild V., De Graaf K., Jager G. (2013). Repeated Exposure More Effective Than Flavour Flavour Learning as Mechanism to Increase Vegetable Consumption in Pre-School Children. Appetite.

[48] Yeomans M.R. (2024). Human Flavour-Based Learning: The Role of Consciousness. Appetite.

[49] Sclafani A., Ackroff K. (2004). The Relationship Between Food Reward and Satiation Revisited. Physiol. Behav..

[50] Beauchamp G.K., Mennella J.A. (2009). Early Flavor Learning and Its Impact on Later Feeding Behavior. J. Pediatr. Gastroenterol. Nutr..

[51] Bouhlal S., Issanchou S., Chabanet C., Nicklaus S. (2014). Just a Pinch of Salt’. An Experimental Comparison of the Effect of Repeated Exposure and Flavor-Flavor Learning with Salt or Spice on Vegetable Acceptance in Toddlers. Appetite.

[52] Brunstrom J.M. (2007). Associative Learning and the Control of Human Dietary Behavior. Appetite.

[53] Eertmans A., Baeyens F., Van den Bergh O. (2001). Food Likes and Their Relative Importance in Human Eating Behavior: Review and Preliminary Suggestions for Health Promotion. Health Educ. Res..

[54] Havermans R.C., Jansen A. (2007). Increasing Children’s Liking of Vegetables Through Flavour-Flavour Learning. Appetite.

[55] Reilly E.E., Anderson L.M., Gorrell S., Schaumberg K., Anderson D.A. (2017). Expanding Exposure-Based Interventions for Eating Disorders. Int. J. Eat. Disord..

[56] Cheah I., Sadat Shimul A., Liang J., Phau I. (2020). Drivers and Barriers Toward Reducing Meat Consumption. Appetite.

[57] Hu F.B., Otis B.O., McCarthy G. (2019). Can Plant-Based Meat Alternatives Be Part of a Healthy and Sustainable Diet?. JAMA.

[58] Röös E., de Groote A., Stephan A. (2022). Meat Tastes Good, Legumes Are Healthy and Meat Substitutes Are Still Strange—The Practice of Protein Consumption Among Swedish Consumers. Appetite.

[59] Singh B., Murphy A., Maher C., Smith A.E. (2024). Time to Form a Habit: A Systematic Review and Meta-Analysis of Health Behaviour Habit Formation and Its Determinants. Healthcare.

[60] Vabø M., Hansen H. (2014). The Relationship Between Food Preferences and Food Choice: A Theoretical Discussion. Int. J. Bus. Soc. Sci..

[61] Emilien C., Hollis J.H. (2017). A Brief Review of Salient Factors Influencing Adult Eating Behaviour. Nutr. Res. Rev..

[62] Olsen A., Ritz C., Hartvig D.L., Møller P. (2011). Comparison of Sensory Specific Satiety and Sensory Specific Desires to Eat in Children and Adults. Appetite.

[63] Scaglioni S., De Cosmi V., Ciappolino V., Parazzini F., Brambilla P., Agostoni C. (2018). Factors Influencing Children’s Eating Behaviours. Nutrients.

[64] Ettinger L., Falkeisen A., Knowles S., Gorman M., Barker S., Moss R., McSweeney M.B. (2022). Consumer Perception and Acceptability of Plant-Based Alternatives to Chicken. Foods.

[65] Løbner M.H., Alexi N., Wilken M.R., Kidmose U. (2022). Forbrugsanalyse for Bælgfrugter.

[66] Bornstein R.F., Kale A.R., Cornell K.R. (1990). Boredom as a Limiting Condition on the Mere Exposure Effect. J. Pers. Soc. Psychol..

[67] Lally P., van Jaarsveld C.H.M., Potts H.W.W., Wardle J. (2010). How Are Habits Formed: Modelling Habit Formation in the Real World. Eur. J. Soc. Psychol..

[68] NHS 5 A Day: What Counts?. https://www.nhs.uk/live-well/eat-well/5-a-day/5-a-day-what-counts/.

[69] Prochaska J.O., Velicer W.F. (1997). The Transtheoretical Model of Health Behavior Change. Am. J. Health Promot..

[70] Steptoe A., Pollard T.M., Wardle J. (1995). Development of a Measure of the Motives Underlying the Selection of Food: The Food Choice Questionnaire. Appetite.

[71] Ventura A.K., Worobey J. (2013). Early Influences on the Development of Food Preferences. Curr. Biol..

[72] Brunstrom J.M. (2005). Dietary Learning in Humans: Directions for Future Research. Physiol. Behav..

[73] Birch L.L., McPhee L., Steinberg L., Sullivan S. (1990). Conditioned Flavor Preferences in Young Children. Physiol. Behav..

[74] Havermans R.C., van den Heuvel E., Guichard E., Salles C. (2023). Acquired Tastes: On the Learning of Human Food Preferences. Flavor.

[75] Mobini S., Chambers L.C., Yeomans M.R. (2007). Effects of Hunger State on Flavour Pleasantness Conditioning at Home: Flavour–Nutrient Learning vs. Flavour–Flavour Learning. Appetite.

[76] Zeinstra G.G., Koelen M.A., Kok F.J., de Graaf C. (2009). Children’s Hard-Wired Aversion to Pure Vegetable Tastes. A ’Failed’ Flavour–Nutrient Learning Study. Appetite.

[77] Attuquayefio T., Parish S., Rogers P.J., Brunstrom J.M. (2020). No Evidence of Flavour-Nutrient Learning in a Two-Week ’Home Exposure’ Study in Humans. Appetite.

[78] Moss R., LeBlanc J., Gorman M., Ritchie C., Duizer L., McSweeney M.B. (2023). A Prospective Review of the Sensory Properties of Plant-Based Dairy and Meat Alternatives with a Focus on Texture. Foods.

[79] Kamei M., Nishibe M., Araki R., Kohyama K., Kusakabe Y. (2024). Effect of Texture Preference on Food Texture Perception: Exploring the Role of Matching Food Texture and Preference. Appetite.

[80] Drewnowski A. (1997). Taste Preferences and Food Intake. Annu. Rev. Nutr..

[81] Scott C.L., Downey R.G. (2007). Types of Food Aversions: Animal, Vegetable, and Texture. J. Psychol..

[82] Zajonc R.B. (2001). Mere Exposure: A Gateway to the Subliminal. Curr. Dir. Psychol. Sci..

[83] Sullivan S.A., Birch L.L. (1990). Pass the Sugar, Pass the Salt: Experience Dictates Preference. Dev. Psychol..

[84] Birch L.L. (1998). Psychological Influences on the Childhood Diet. J. Nutr..

[85] Modlinska K., Adamczyk D., Maison D., Pisula W. (2020). Gender Differences in Attitudes to Vegans/Vegetarians and Their Food Preferences, and Their Implications for Promoting Sustainable Dietary Patterns—A Systematic Review. Sustainability.

