# Supplementary material for: A Comparison of Mere-Exposure, Flavor–Flavor and Flavor–Nutrient Learning Strategies in Enhancing Legume Preferences in an Adult Sample
Source: Foods. 2026 Aug 18;15(16):2884. doi: 10.3390/foods15162884 (PMC13511802; doi:10.3390/foods15162884)
Supplement: Supplementary file 1 [file foods-15-02884-s001.zip › foods-4455441-supplementary.pdf]

Supplementary file S1: Full questionnaire overview

The full questionnaire overview (developed English version).

Section	Question	Answer format
**Introduction**	Thank you for considering participating in this research study. By continuing you are giving consent to participate in this study under the aforementioned conditions.<br><br>GDPR text inserted here.	Categories, single choice:<br>- I confirm to have read and understood the conditions for participation in the study and I give consent to the use of my responses, for data analysis and publication.<br>- I do not wish to participate in the study.
**Control questions**	Do you live in Denmark?/ China?	Categories:<br>Yes/ No.<br><br>If No, they would be sent to the end of the questionnaire.
	How old are you?	Numeric, open reply field.<br><br>If under 18, they would be sent to the end of the questionnaire.
	Do you have any psychological or physical illnesses that affect your food intake?	Categories, single choice:<br>Yes/ No.<br><br>If Yes, they would be sent to the end of the questionnaire.
	Are you vegan or vegetarian?	Categories, single choice:<br>Yes/ No.<br><br>If Yes, they would be sent to the end of the questionnaire.
**Questions that were repeated for legumes, plant-based meat analogues and hybrids, and cell-based foods.**<br><br>**Incl. definitions of each.**<br><br>**Note: The order of the food categories was randomized.**	Which of the following examples of **legumes** are you familiar with eating? Please, choose all of the types you are familiar with eating.<br><br>Definition: A foodstuff comprising the edible seeds or fruits (pods) of a leguminous plant, as beans, peas, lentils, etc. Ref: legume, n. meanings, etymology and more | (Oxford English Dictionary, n.d)	Categories, CATA:<br>- Chickpeas<br>- Green peas<br>- Fava beans<br>- Green lentils<br>- Red lentils<br>- Yellow split peas<br>- Black beans<br>- Lima beans<br>- Soy beans<br>- Edamame beans<br>- Peanuts<br>- Not familiar with any of the mentioned examples

	Overall, how much do you like **legumes**? Answer this based on your overall and immediate opinion of legumes.	9 pt. hedonic scale:<br>1. Do not like at all –<br>9. Like extremely much
	In your opinion, how do you see a potential intake of **legumes**? Please, mark on the scale how you see an intake of legumes for the following factors.<br>One click is sufficient.	10 cm VAS:<br>- Unwise - Wise<br>- Uncomfortable - Enjoyable<br>- Boring - Exciting<br>- Repulsive - Attractive<br>- Unnecessary - Necessary<br>- Non-nutritious - Nutritious<br>- Unhealthy - Healthy<br>- Tasteless - Tasty<br>- Inexpensive - Expensive<br>- Artificial - Natural<br>- Harmful to climate – Climate-friendly<br>- Unsafe to eat - Safe to eat<br>- Unknown long-term consequences - Known long-term consequences
	In which of the **following meal situations** do you find it appropriate to consume **legumes**? Please, choose all the appropriate situations.	Categories, CATA:<br>- Breakfast<br>- Lunch<br>- Dinner<br>- As a Snack<br>- Never appropriate
	In which of the following **physical environments** do you find it appropriate to consume **legumes**? Please, choose all the appropriate environments.	Categories, CATA:<br>- On the Go<br>- As Take-away<br>- At Home<br>- In Restaurants<br>- In Cafés<br>- In Canteens<br>- At Friends/ families houses<br>- Never appropriate
	In which of the following **social situations** do you find it appropriate to consume **legumes**? Please, choose all the appropriate social situations.	Categories, CATA:<br>- With Friends<br>- With Adult family members<br>- With Child family members<br>- With Classmates/ colleagues<br>- Alone<br>- Never appropriate
	How often do you consume **legumes**? Choose the option that most closely resembles your consumption pattern.	Categories, single choice:<br>- Never<br>- Less than once a week<br>- Once a week

		- 2 - 6 times a week<br>- Daily
	Which of the following examples of **plant-based meat analogues and hybrids** are you familiar with eating? Please, choose all of the types you are familiar with eating.<br><br>Definition: Plant-based meat analogues refer to vegetable proteins, such as pulses, wheat gluten and soy protein, which are processed into meat-like products. These products approximate certain qualities, such as texture, flavor, and color, and nutritional characteristics of specific types of meat. Hybrid meat products contain varying amounts of plant-based ingredients (from 25% - 50%).<br>Examples: mince, burger patties in both fully plant-based and hybrid form (Grasso & Goksen, 2023).	Categories, CATA:<br>- Plant-based mince<br>- Plant-based sausages<br>- Plant-based cold cuts<br>- Hybrid mince (meat- and plant-based)<br>- Ready-made meals<br>- Plant-based meat balls<br>- Plant-based burger patties<br>- Hybrid burger patties (meat and plant-based)<br>- Hybrid cold cuts (meat and plant-based)<br>- Not familiar with any of the mentioned examples
	Overall, how much do you like **plant-based meat analogues and hybrids**? Answer this based on your overall and immediate opinion of plant-based meat analogues.	9 pt hedonic scale:<br>1. Do not like at all –<br>9. Like extremely much
	In your opinion, how do you see a potential intake of **plant-based meat analogues and hybrids**? Please, mark on the scale how you see an intake of plant-based meat analogues for the following factors.<br>One click is sufficient.	10 cm VAS:<br>- Unwise - Wise<br>- Uncomfortable - Enjoyable<br>- Boring - Exciting<br>- Repulsive - Attractive<br>- Unnecessary - Necessary<br>- Non-nutritious - Nutritious<br>- Unhealthy - Healthy<br>- Tasteless - Tasty<br>- Inexpensive - Expensive<br>- Artificial - Natural<br>- Harmful to climate – Climate-friendly

		- Unsafe to eat - Safe to eat<br>- Unknown long-term consequences - Known long-term consequences
	In which of the following **meal situations** do you find it appropriate to consume **plant-based meat analogues and hybrids**? Please, choose all the appropriate situations.	Categories, CATA:<br>- Breakfast<br>- Lunch<br>- Dinner<br>- As a snack<br>- Never appropriate
	In which of the following **physical environments** do you find it appropriate to consume **plant-based meat analogues and hybrids**? Please, choose all the appropriate environments.	Categories, CATA:<br>- On the Go<br>- As Take-away<br>- At Home<br>- In Restaurants<br>- In Cafés<br>- In Canteens<br>- At Friends/ families houses<br>- Never appropriate
	In which of the following **social situations** do you find it appropriate to consume **plant-based meat analogues and hybrids**? Please, choose all the appropriate social situations.	Categories, CATA:<br>- With Friends<br>- With Adult family members<br>- With Child family members<br>- With Classmates/colleagues<br>- Alone<br>- Never appropriate
	How often do you consume **plant-based meat analogues and hybrids**? Choose the option that most closely resembles your consumption pattern.	Categories, single choice:<br>- Never<br>- Less than once a week<br>- Once a week<br>- 2 - 6 times a week<br>- Daily
	Which of the following examples of **cell-based foods** are you familiar with? Please, choose all of the types that you have heard of.<br><br>Definition: Cell-based food production involves using cells isolated from animals, plants or microorganisms to produce food products, ingredients or additives. Often the products can be seen as comparable to the existing animal products, such as meat, poultry, fish and	Categories, CATA:<br>- Red meat<br>- Poultry<br>- Fish, shellfish<br>- Eggs<br>- Dairy products

	shellfish, dairy products and eggs (Food and Agriculture Organization of the United Nations, 2023b, 2023a).	
	Overall, how much do you like **cell-based foods**? Answer this based on your overall and immediate opinion of cell-based foods.	9 pt hedonic scale:<br>1. Do not like at all –<br>9. Like extremely much
	In your opinion, how do you see a potential intake of **cell-based foods?** Please, mark on the scale how you see an intake of cell-based foods for the following factors. One click is sufficient.	10 cm VAS:<br>- Unwise - Wise<br>- Uncomfortable - Enjoyable<br>- Boring - Exciting<br>- Repulsive - Attractive<br>- Unnecessary - Necessary<br>- Non-nutritious - Nutritious<br>- Unhealthy - Healthy<br>- Tasteless - Tasty<br>- Inexpensive - Expensive<br>- Artificial - Natural<br>- Harmful to climate – Climate-friendly<br>- Unsafe to eat - Safe to eat<br>- Unknown long-term consequences - Known long-term consequences
	In which of the following **meal situations** do you find it appropriate to consume **cell-based foods**? Please, choose all the appropriate situations.	Categories, CATA:<br>- Breakfast<br>- Lunch<br>- Dinner<br>- As a snack<br>- Never appropriate
	In which of the following **physical environments** do you find it appropriate to consume **cell-based foods**? Please, choose all the appropriate environments.	Categories, CATA:<br>- On the Go<br>- As Take-away<br>- At Home<br>- In Restaurants<br>- In Cafés<br>- In Canteens<br>- At Friends/ families houses<br>- Never appropriate
	In which of the following **social situations** do you find it appropriate to consume **cell-**	Categories, CATA:<br>- With Friends<br>- With Adult family members<br>- With Child family members

	**based foods**? Please, choose all the appropriate social situations.	- With Classmates/colleagues<br>- Alone<br>- Never appropriate
**Personal relation to sustainable eating behavior**	Please, indicate the statement that best aligns with your current stage of sustainable eating behavior practices.	Categories, single choice:<br>Stage 1: I have no intention of a sustainable eating behavior change.<br>Stage 2: I am aware of the needed sustainable eating behavior change but have not yet committed to change.<br>Stage 3: I have begun planning to make sustainable eating behavior changes and am committed to following through.<br>Stage 4: I have implemented the intended sustainable eating behavior modifications in an effort to change behavior.<br>Stage 5: I am maintaining sustainable eating behavior change and trying to prevent termination of behavior change.<br>Stage 6: I have failed to maintain the intended sustainable eating behavior change.
	How important is it to you that your food is climate friendly?	10cm VAS:<br>1 Not at all important –<br>10 Extremely important
	To what extent are these values important as 'guiding principles' in your life?<br>Please carefully consider the values and do not mark more than 2 as 'Extremely important'.	9 pt. Scale:<br>(negative 1) - 1: Opposed to my values<br>0: Not important<br>7: Extremely important<br><br>Biospheric Factors:<br>- Preventing pollution<br>- Respecting the earth<br>- Unity with nature<br>- Protecting the environment
**Demographic questions**	Which of the following diets is the closest to yours?	Categories, single choice:<br>- Omnivore: Diversified diet including plant-based foods, meat, dairy products, eggs, seafood and other foods.<br>- Flexitarian: Eating a mostly

		plant-based diet including dairy and egg, occasionally fish and/or meat products.<br>- Pescetarian: Consumes fish, egg and dairy but no other animal products.<br>- Other
	How many people in total, including yourself, are in your household?	Numeric, open reply field.
	How many children are there in your household?	Numeric, open reply field.
	What is the highest level of education that you have completed?	Categories, single choice:<br>- Elementary School<br>- Junior Highschool<br>- Highschool<br>- Short undergraduate (2 Years)<br>- Undergraduate Degree (3 – 4 Years)<br>- Master's Degree<br>- Doctoral Degree<br>- Other/ Does not know
	What gender do you identify as?	Categories, single choice:<br>- Female<br>- Male<br>- Other
	If you have any comments, feel free to write them here	Open reply field, text.
**Conclusion**	Thank you very much for your time and participation in the questionnaire.	
